# Optical Flow Cell for Measuring Size, Velocity and Composition of Flowing Droplets

**DOI:** 10.3390/mi8020058

**Published:** 2017-02-16

**Authors:** Sammer-ul Hassan, Adrian M. Nightingale, Xize Niu

**Affiliations:** 1 Faculty of Engineering and the Environment, University of Southampton, Southampton SO17 1BJ, UK; S.Hassan@soton.ac.uk (S.H.); a.nightingale@soton.ac.uk (A.M.N.); 2 Institute for Life Sciences, University of Southampton, Southampton SO17 1BJ, UK

**Keywords:** droplet microfluidics, absorbance, real-time monitoring, velocity measurement, enzymatic assays, optical detections

## Abstract

Here an optical flow cell with two light paths is reported that can accurately quantify the size and velocity of droplets flowing through a microchannel. The flow cell can measure the time taken for droplets to pass between and through two conjoined light paths, and thereby is capable of measuring the velocities (0.2–5.45 mm/s) and sizes of droplets (length > 0.8 mm). The composition of the droplet can also be accurately quantified via optical absorption measurements. The device has a small footprint and uses low-powered, low-cost components, which make it ideally suited for use in field-deployable and portable analytical devices.

## 1. Introduction

Droplet-based microfluidics has emerged as an effective technique to manipulate sample fluids and analyze sample droplets in a high-throughput format. Samples are compartmentalized in nanoliter-sized droplets which eliminate dispersion or band-broadening contrary to the continuous microfluidics [1]. The accurate and precise measurement of flow rates is crucial for high-throughput applications such as flow cytometry, particle counting, enzyme kinetics and cell sorting [2]. In particular, droplets traveling in the microchannels require the detection of droplet position, size, and velocity for real-time manipulation. Several studies have explored enzyme kinetics in continuous droplet streams using multi-point detection [3,4]. These studies rely on accurate control of flow velocity and droplet size, because any fluctuation of the flow rate could affect the characterization of the rates of the reaction, which is detrimental to the accurate measurement of Michaelis–Menten kinetics.

Electrical and optical detection are the most common methods for the detection of droplets. The electrical method of droplet detection involves the detection of the contrast present between the oil and aqueous droplet due to the differences in their electrical properties. Electrochemical and capacitive-based detection methods have been used widely. Electrochemical detections [5] work on the principle of interaction between analytes and electrodes while capacitive sensing works via detecting the change in capacitance between two electrodes produced by the changing dielectric constant of oil and water. Electrochemical detections only distinguish analytes that are electroactive, and the electrodes require frequent calibrations after short intervals because the electrodes are prone to changes in pH, ionic concentration and temperature [5,6,7,8]. Moreover, electrochemical detection requires droplets to be in contact with the electrodes, but a thin oil layer can separate the droplet from the electrode and limits the signal. Droplets can be wetted on the surface of the electrode, but the wetting can lead to contamination of the sample between the droplets [9].

Capacitive detections offer the detection of droplets without the requirement of contacting the electrodes. Several capacitance-based detectors have been developed for the detection of droplet position, size, and velocity [9,10,11,12,13,14]. For example, Chen et al. [12] developed the miniaturized capacitance-based sensor for the detection of droplet position, size, composition and percentage water uptake for hygroscopic liquids. Elbuken et al. [9] presented a capacitance-based system for detecting the presence, size and speed of droplets using commercially available, inexpensive capacitive sensors. Niu et al. [11] reported the design and implementation of capacitive detection and control of droplets using integrated microfluidic and control circuits to monitor in situ the volume, the velocity and even the composition of the droplets and sorting of droplets in real time.

Optical detection methods have been developed to analyze the size and velocity of droplets, mostly based on the analysis of videos recorded by high-speed cameras [15,16]. While this approach can accurately measure droplet sizes and velocities over a wide range, the need for bulky hardware (e.g., high-speed camera, microscope, high-specification computing hardware) means that it cannot be used in portable point-of-care applications. Several other optical methods have been reported to overcome these challenges such as by Jakiela et al. [17], who reduced the data storage requirement and performed the real-time detection of droplet velocity and size by imaging the center-line of the microchannel. Revellin et al. [18] used two fibers at a fixed distance and photodiodes on the opposite sides to measure the mean velocity and length of the slugs (gas bubbles). Similarly, Vincent et al. [15] developed an optical detector using two photodiodes to measure the velocity, length, and frequency of droplets. The real-time measurement of droplet characteristics was performed by collecting a differential signal from illumination observed during the passage of the droplets. Apart from the illumination, a Doppler-based optical method [2] has also been developed to detect the flow velocities of the droplet flow. The authors detected the Doppler shift from the scattered light while the droplet passed through an illumination source.

However, the systems mentioned above were bulkier and had not been miniaturized for portable systems. This study reports an optical flow cell to quantify the size and velocity of droplets flowing inside microchannels. The working principle of the flow cell involves the measurement of the signal from two light paths and is analogous to a previously reported capacitive-based device [11].

## 2. Materials and Methods

### 2.1. Materials and Reagents

Commercially available reagents were bought from Sigma-Aldrich (Dorset, UK) and used without further purification. Glucose oxidase, horseradish peroxidase (HRP), 4-aminoantipyrine, and phenol were prepared in 0.1 M phosphate buffered saline (PBS) at pH of 7.4. PBS was prepared by dissolving Tablets (Sigma-Aldrich) in ultrapure water (18.2 MΩ·cm, MilliQ, Watford, UK). The final reagent concentrations in the mixture were as follows: glucose oxidase (30 U·mL^−1^), peroxidase (30 U·mL^−1^), 4-aminoantipyrine (1.54 mM) and phenol (22 mM). Standard solutions of glucose sample were prepared by dissolving D-glucose in 0.1 M PBS. Red food dye solutions were prepared by dissolving powder (East End Foods plc, West Bromwich, UK) in DI water.

### 2.2. Fabrication of Microchip

The T-junction chip used to generate droplets was fabricated as previously shown by Hassan et al. [4]. Briefly, a mould was designed in SolidWorks (Dassault Systemes, Vélizy-Villacoublay, France) and printed using an Objet500 Connex3 3D printer (Stanford Marsh Ltd., Worcester, UK). The mould was cleaned, dried in an oven overnight at 60 °C and treated with Aquapel (PPG Industries, Pittsburgh, PA, USA) to render the surface of the mould hydrophobic and aid removal of the polydimethylsiloxane (PDMS) chip after casting. Silicone elastomer Base (Sylgard 184, Dow corning, Seneffe, Belgium) was mixed with the curing agent (Dow Corning) in a clean petri dish, and the ratio was kept at 10:1 (*w*/*w*), respectively. The mixture was then added onto the mould to cast channel layer and was then left in the oven for 2 h at 60 °C. The PDMS layer was removed from the mould with all the channels transferred to the PDMS surface and the holes were punched for liquid inlets and outlet. The PDMS layer with channels was then bonded to another flat piece of PDMS using the “half-cure” method [19] and left in the oven to fully cure for 4 h at 60 °C. The channels were flushed with Aquapel to render the channels hydrophobic and used for droplet generation.

### 2.3. Droplet Generation

Two inlets were used to inject aqueous samples, and the third inlet was used to pump carrier fluid (FC-40 fluorinated oil, 3M, Bracknell, UK) with 0.35% *w*/*w* surfactant (non-ionic tri-block copolymer surfactant) synthesized in-house [20]. Initially, the red dye and water were injected to the aqueous inlets to generate red droplets (mixing volume ratio was 1:1). Glucose reagents and samples were also injected into the aqueous inlets to generate glucose reaction droplets. All fluids were pumped via Syringe pumps (PHD 2000, Harvard Apparatus, Cambridge, UK) and the velocity and droplet lengths were altered by varying oil and aqueous flow rates. Polytetrafluoroethylene (PTFE) tubing (0.3 mm ID, 0.5 mm OD, Adtech Polymer Engineering Ltd., Gloucester, UK) was glued on the outlet, and the droplets were transported to the flow cell for optical detections.

### 2.4. Fabrication of the Flow Cell

**Figure 1:**
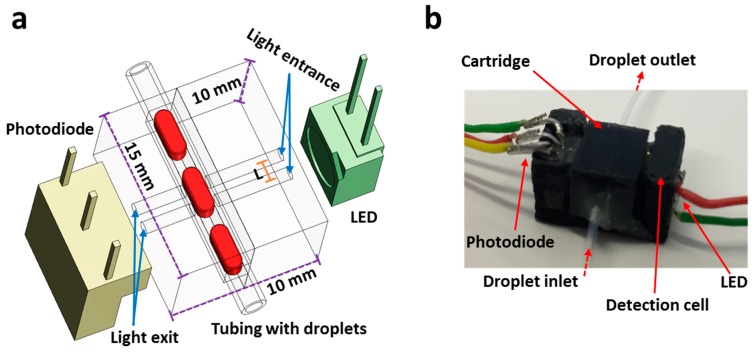
Flow cell: (**a**) Schematic of the optical flow cell showing two holes for light entrance to the channel and light exit to the detector. *L* is a center-to-center distance between two light paths; (**b**) Snapshot of the fully assembled flow cell.

A 3D schematic of the flow cell is shown in Figure 1a. This miniaturized flow cell with dual light paths was designed using SolidWorks and AutoCAD. The flow cell consists of two interlocking structures, a cartridge, and a detection cell [4]. The cartridge is composed of two interlocking micromilled polymethylmethacrylate (PMMA) pieces with a channel cut into one piece to hold 0.5 mm OD tubing (0.3 mm ID). The detection cell houses the cartridge, light-emitting diode (LED), and photodiodes (Figure 1b) and allows accurate alignment of the optical components. Once fully assembled, the flow cell measures only 10 mm × 10 mm × 15 mm, therefore, it can be easily accommodated within a field-deployable point-of-care (POC) device.

The detection cell was printed in black poly(lactic acid) (PLA) material using an Ultimaker-2 3D printer (Ultimaker BV, Geldermalsen, The Netherlands). The cartridge was fabricated by a precision micromilling of PMMA sheet (black) using LPKF Protomat S100 micromilling machine (LPKF Laser & Electronics Ltd., Berkshire, UK) as previously detailed [21]. Briefly, the channels for holding tubing were micromilled with a 500-μm-diameter end milling bits (LPKF Laser & Electronics Ltd., Berkshire, UK), and the holes were drilled with a 200-μm-diameter drilling bit (center-to-center distance (*L*) = 0.5 mm) as shown in schematic in Figure 1. Another PMMA piece was also fabricated with similar dimensions and holes, and both the parts were glued (Super Glue, Loctite, Hertfordshire, UK) together using alignment pins with the tubing inserted into the channels. LED (ASMT-QGBE-NFH0E, Avago Technologies, Cambridge, UK) and photodetector (TSL257, Texas Advance Optical Solutions, Cambridge, UK) were fixed in the detection cell.

### 2.5. Data Processing

The voltage readout from photodiode was displayed on a computer using LabVIEW (National Instruments, Austin, TX, USA) via a microcontroller (Arduino Nano, New York, NY, USA) for data collection by. The modified version of the Beer-Lambert law [4] was used to measure the absorption values for the droplets. Briefly, the equation allows for small changes in the light intensity of the LED via the addition of light intensity levels for carrier oil FC-40. The composition of the FC-40 does not change during measurements of blanks and samples; therefore the light intensity of oil can be used to adjust for any variations in the droplet levels caused by variations in LED light intensity.

Videos of droplets were recorded at a tubing outlet of the flow cell using a handheld microscope (GX Microscopes, Suffolk, UK) fixed on a stage. These recorded videos were analyzed by ImageJ to measure the flow rate and droplet lengths.

## 3. Results and Discussion

### 3.1. Operation of the Flow Cell

**Figure 2:**
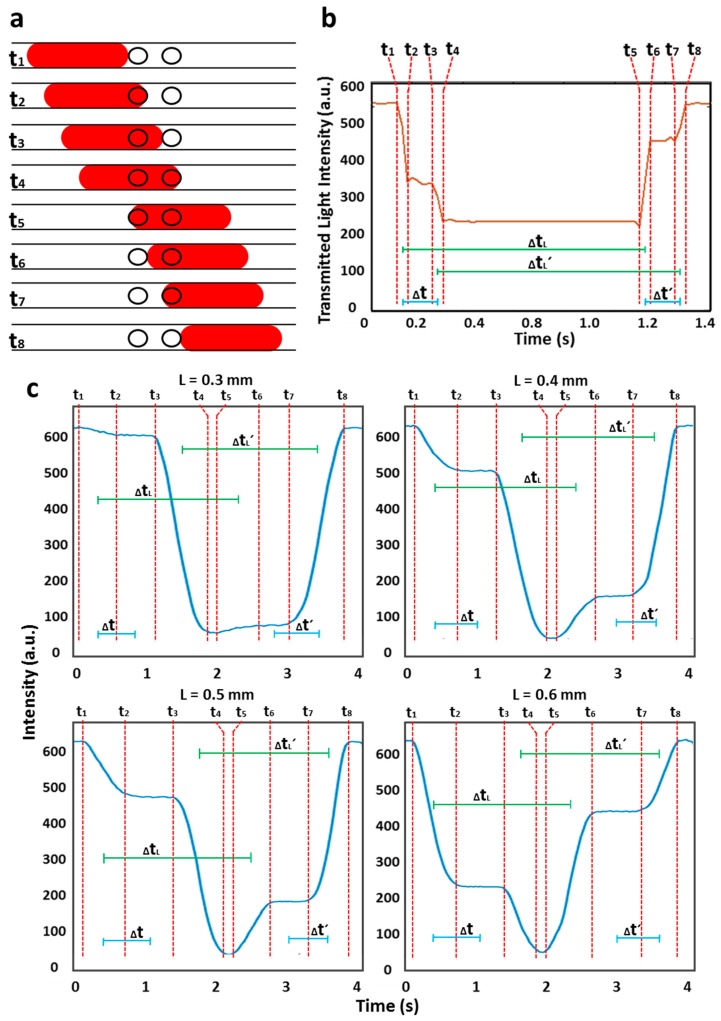
Working principle of the optical flow cell: (**a**) Schematic of the device; (**b**) Plot of transmitted light intensity over time as droplet travels through the holes. The times *t*_1_ → *t*_8_ relate to the moment when droplet enters and exits the holes; (**c**) The graphs showing a drop in intensity levels during the passage of droplets (droplet velocity and length were 1.65 mm/s and 0.94 mm, respectively) from light paths of different distances (*L* = 0.3, 0.4, 0.5, and 0.6 mm).

As shown in Figure 1, light emitted from the LED shines on the droplets in a tubing, and transmitted light reaches the photodiode. Two holes with a 0.2 mm ID were micromilled in the cartridge with a fixed distance of 0.5 mm (center-to-center distance referred to as *L*) and aligned. The diameter of the holes was deliberately chosen to be smaller than the inner diameter of the tubing to ensure that all light traveled through the fluid and to reduce the lensing effect of the curved tubing walls. The working principle of the flow cell is shown in Figure 2. As droplets pass through each separate light path, the transmitted light received by the photodiode varies due to the combined effects of refractive index change and absorbance by the droplet contents. As each droplet enters the light path, the transmitted light intensity drops from a higher value (corresponding to light passing through the carrier oil) to a lower value (*t*_1_ → *t*_2_), as shown in Figure 2a. The intensity value of the droplet stays constant (*t*_2_ → *t*_3_) and further drops when the droplet passes through the second light path (*t*_3_ → *t*_4_). The droplet intensity level stays the same while the droplet remains under both light paths (*t*_4_ → *t*_5_). Similarly, the intensity level increases when the droplet exits the first light path (*t*_5_ → *t*_6_), stays constant (*t*_6_ → *t*_7_) and returns to its initial high level when the droplet exits both light paths (*t*_7_ → *t*_8_). Defining ∆t as the time taken by a droplet to travel between two light paths, *L* as the distance between two light paths and ∆*t_L_* as the time required for a droplet to pass through a light path (see Figure 2b), the velocity, *v*, of the droplet is given by *L*/∆*t* and the droplet length by *v*∆*t_L_*. Similarly, *L*/∆*t*′ and *v*∆*t_L_*′ are also used to measure the velocity and size of the droplet, respectively. Note that the droplet intensity for entering and exiting the light path was not equal which can be attributed to the slight misalignment of the 0.2 mm holes or the position of the holes in reference to the photodiode. Nonetheless, this difference in intensity level is less important in accurately measuring the size and velocity of the droplets.

**Figure 3:**
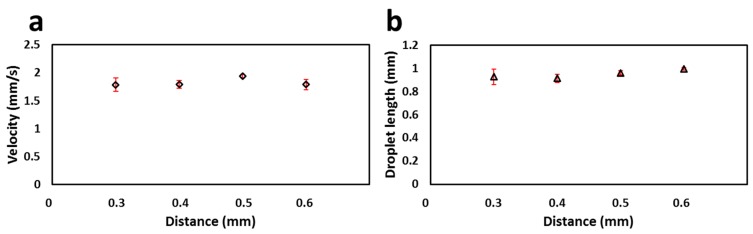
Droplet velocity and length measurements in dual light paths with different distances (*L* = 0.3, 0.4, 0.5, and 0.6 mm): (**a**) Velocity measured by flow cell with a dual light path using different *L* values; (**b**) Droplet length measured by the flow cell using different *L* values. Both droplet velocity (1.65 mm/s) and length (0.94 mm) were measured to be similar with %RSD values of 3% and 4% respectively.

We initially characterized the distance between the holes (*L* = 0.3, 0.4, 0.5, and 0.6 mm) in the cartridge by flowing droplets of red food dye at a fixed flow rate and droplet length as shown in Figure 2c. It can be seen from the intensity signal that ∆*t* decreased with a decrease in *L* values at a fixed flow rate. The droplet velocity and length were measured from the intensity plots shown in Figure 2c. The %RSD of the droplet velocity (1.65 mm/s) and droplet length (0.94 mm) for each *L* value was found to be 3% and 4%, respectively (Figure 3a,b). This low error proves that the velocity and length of droplets do not change with a change in *L* values, but the *L* value of 0.3 mm was found to be too small to clearly observe the sharp drop in intensity. Hence, in later experiments, the *L* value of 0.5 mm was used. There is no limit for the larger *L* values but increasing the *L* value requires larger plugs to be injected into the flow cell.

### 3.2. Calibration of the Flow Cell

**Figure 4:**
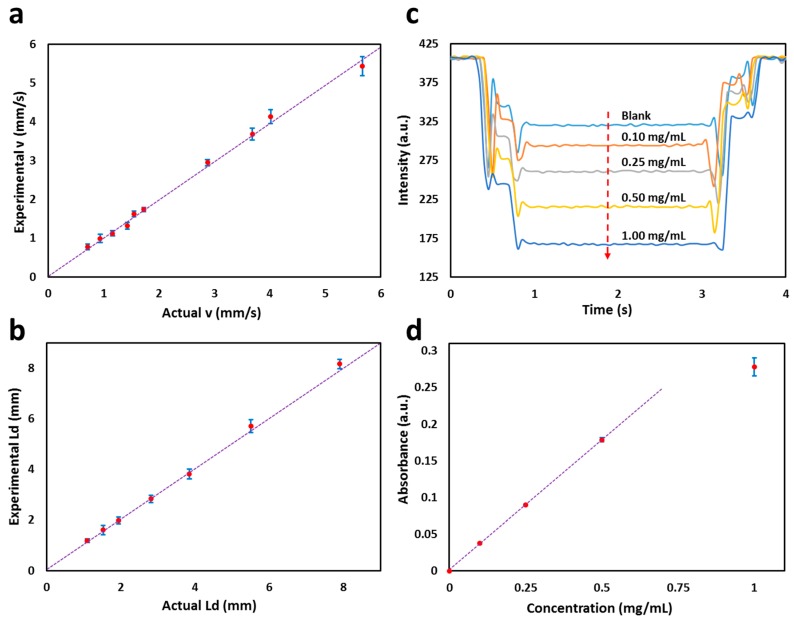
Characterization of the flow cell: (**a**) Experimental velocity vs. actual velocity; (**b**) Experimental droplet length vs. actual droplet length. The dotted line shows the 1:1 equivalence; (**c**) Transmitted light intensity response of blank (pure water) and red food dye concentrations (0.10, 0.25, 0.50, and 1.00 mg/mL) as a voltage output from the photodiode. The arrow shows the increase in concentration of the red food dye; (**d**) Plot of absorbance against droplets containing red food dye concentration measured in the flow cell with dual light paths. Droplet absorbance against dye concentration. A line of best fit (*R*^2^ = 0.999) shows the linear response for absorbance values <0.2.

To test the performance of the flow cell, droplets of different velocities (0.77, 0.99, 1.12, 1.32, 1.62, 1.74, 2.95, 3.68, 4.13, and 5.44 mm/s) and lengths (1.10, 1.53, 1.94, 2.82, 3.85, 5.51, and 7.90 mm) were generated, measured using the flow cell and then compared with actual values obtained from recorded videos. As shown in Figure 4a,b, there was excellent agreement between the two methods, with errors for the optical flow cell relative to the video results of <6% for droplet velocity and <5% for droplet size. The flow cell was capable of measuring the velocities of 0.2–5.45 mm/s and droplet lengths of >0.8 mm. The range of the droplet velocity and size detection with the flow cell depends on the hole and tubing diameter and the center-to-center distance (*L*). Higher velocities and smaller droplets can also be detected by decreasing the hole and tubing diameters and L values.

A key advantage of this optical flow cell over other previously reported devices [2,9,11,15] is that it can additionally be used for accurate measurement of droplet composition via absorption spectrophotometry. The flow cell was characterized by flowing droplets of blank composed of pure water and red food dye at different concentrations (0.10, 0.25, 0.50, and 1.00 mg/mL) through the flow cell and measuring the response as a voltage output from the photodiode as shown in Figure 4c. As the dye concentration in the droplets dropped from high to low, the magnitude of the intensity drop also decreased. The absorbance of each concentration of the droplets was calculated against a set of blank droplets. Each concentration was repeated three times and the overall %RSD was found to be ~2%. Figure 4d shows the measured absorbance values versus dye concentration.

### 3.3. Glucose Colorimetric Assay

**Figure 5:**
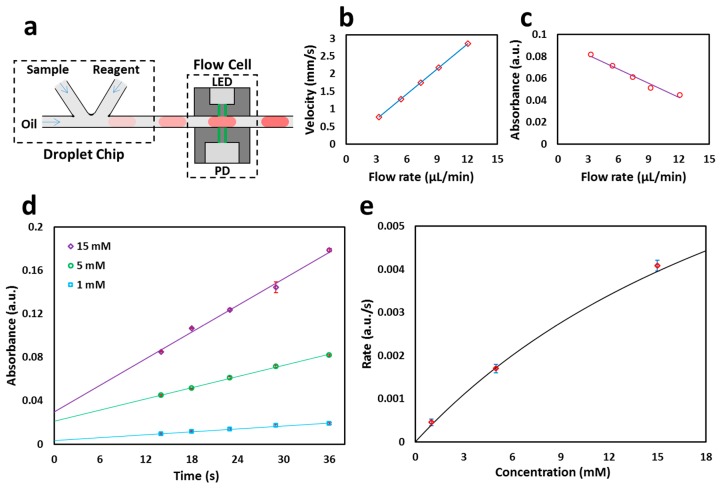
Enzymatic glucose reaction in droplets at different velocities: (**a**) Schematic of the droplet generation and injection into the flow cell; (**b**) Change in velocity versus total flow rate change. Each point corresponds to velocities of 0.77, 1.28, 1.75, 2.17 and 2.85 mm/s and flow rates of 3.3, 5.4, 7.4, 9.2, and 12.1 µL/min at a distance of 3.8 cm; (**c**) Absorbance of 5 mM glucose decreases with increase in flow rate as the glucose reaction droplet reaches the detector in shorter times; (**d**) The absorbances of glucose droplets (1, 5, 15 mM) plotted as lines of best fit against time; (**e**) The data is fitted by nonlinear regression with a Michaelis–Menten curve. *V*_max_ = 0.0068 a.u./s and *K*_m_ = 10.77 mM.

Having calibrated the flow rate sensor using food dye, a colorimetric assay was implemented to quantify glucose concentrations in sample solutions. Figure 5a shows the schematic of the quantification of the progress of a colorimetric glucose assay over time by placing the flow cell at a set distance (3.8 cm) downstream of a T-junction generating droplets. The total flow rate was varied, the linear flow rate was quantified inline using the optical flow cell (found to be 0.77, 1.28, 1.75, 2.17 and 2.85 mm/s) and then it was used to calculate the reaction times (14, 18, 23, 29, and 36 s). The flow rates were calculated to be 3.3, 5.4, 7.4, 9.2, and 12.1 µL/min. Figure 5b shows the relationship between the flow rate and the velocity measured via the flow cell. The droplets composed of 1, 5, and 15 mM glucose and glucose-specific colorimetric reagents (containing glucose oxidase, HRP, phenol, and 4-aminoantipyrine) were generated in a stream of fluorous oil. Blank measurements were taken by using zero concentration glucose solution (and reagents) and used to calculate the absorbance of the glucose-containing droplets. As the flow rate increased, the absorbance of the glucose reaction droplets decreased due to the droplets reaching the detectors in shorter times (Figure 5c). The absorbance was seen to increase linearly with the reaction time as expected and shows that the flow cell with dual light paths can be utilized for quantitative chemical assaying (Figure 5d). The rates of the glucose reaction were calculated by measuring the slope of the linear best fit.

The obtained reaction rates were then plotted against the glucose concentration and fitted with a Michaelis–Menten curve using nonlinear regression (Figure 5e). The curve fits the data exceptionally well (*R*^2^ ≈ 0.998) with *V*_max_ = 0.0068 a.u./s and *K*_m_ = 10.77 mM. This *K*_m_ value is in agreement with previously reported values (7.63 ± 2.22 mM, 6.47 ± 0.85 mM) for the same assay under similar reaction conditions [22]. The limit of detection (3 × standard deviation of blank measurements) was found to be 0.1 mM, similar to that of conventional benchtop methods (e.g., NZYTech D-Glucose GOD-POD colorimetric assay kit, limit of detection 0.06 mM).

## 4. Conclusions

This paper presents the development of an optical flow cell for the accurate measurement of the length, velocity, and composition of droplets. The optical flow cell was fabricated with dual light paths, analogous to a previously reported capacitive-based device [11]. The flow cell was capable of measuring droplet velocities with errors of <6% (range 0.2–5.45 mm/s) and lengths with errors of <5% (minimum length 800 µm), with the operational ranges extendable by controlling the device dimensions. We envisage the flow cell could be used as a miniaturized device for the simultaneous detection of droplets and the measurement of colorimetric reactions within droplets. The small footprint of the flow cell makes it an ideal component to be integrated in a portable or wearable microfluidics device for point-of-care diagnostic and monitoring.

## References

[1] Song H., Tice J.D., Ismagilov R.F. (2003). A Microfluidic system for controlling reaction networks in time doppler-based flow rate sensing in microfluidic channels. Angew. Chem. Int. Ed..

[2] Stern L., Bakal A., Tzur M., Veinguer M., Mazurski N., Cohen N., Levy U. (2014). Doppler-based flow rate sensing in microfluidic channels. Sensors.

[3] Sjostrom S.L., Joensson H.N., Svahn H.A. (2013). Multiplex analysis of enzyme kinetics and inhibition by droplet microfluidics using picoinjectors. Lab Chip.

[4] Hassan S., Nightingale A.M., Niu X. (2016). Continuous measurement of enzymatic kinetics in droplet flow for point-of-care monitoring. Analyst.

[5] Pires N.M.N., Dong T., Hank U., Hoivik N. (2014). Recent developments in optical detection technologies in lab-on-a-chip devices for biosensing applications. Sensors.

[6] Wanh J. (2008). Electrochemical glucose biosensors. Chem. Rev..

[7] Mitton K.P., Trevithick J.R. (1994). High-performance liquid chromatography-electrochemical detection of antioxidants in vertebrate lens: Glutathione, tocopherol, and ascorbate. Methods Enzymol..

[8] Wongkaew N., He P., Kurth V., Surareungchai W., Baeumner A.J. (2013). Multi-channel PMMA microfluidic biosensor with integrated IDUAs for electrochemical detection. Anal. Bioanal. Chem..

[9] Elbuken C., Glawdel T., Chan D., Ren C.L. (2011). Detection of microdroplet size and speed using capacitive sensors. Sens. Actuators A Phys..

[10] Dong T., Barbosa C. (2015). Capacitance variation induced by microfluidic two-phase flow across insulated interdigital electrodes in lab-on-chip devices. Sensors.

[11] Niu X., Zhang M., Peng S., Wen W., Sheng P. (2007). Real-time detection, control, and sorting of microfluidic droplets. Biomicrofluidics.

[12] Chen J.Z., Darhuber A.A., Troian S.M., Wagner S. (2004). Capacitive sensing of droplets for microfluidic devices based on thermocapillary actuation. Lab Chip.

[13] Demori M., Ferraria V., Strazzab D., Poesio P. (2010). A capacitive sensor system for the analysis of two-phase flows of oil and conductive water. Sens. Actuators A Phys..

[14] Ernst A., Streule W., Schmitt N., Zengerle R., Koltay P. (2009). A capacitive sensor for non-contact nanoliter droplet detection. Sens. Actuators A Phys..

[15] De Saint Vincent M.R., Cassagnre S., Plantard J., Delville J.P. (2012). Real-time droplet caliper for digital microfluidics. Microfluid. Nanofluid..

[16] Ferraro D., Champ J., Teste B., Serra M., Malaquin L., Viovy J.-L., de Cremoux P., Descroix S. (2016). Microfluidic platform combining droplets and magnetic tweezers: Application to HER2 expression in cancer diagnosis. Sci. Rep..

[17] Jakiela S., Makulska S., Korczyk P.M., Garstecki P. (2011). Speed of flow of individual droplets in microfluidic channels as a function of the capillary number, volume of droplets and contrast of viscosities. Lab Chip.

[18] Revellina R., Dupontb V., Ursenbachera T., Thomea J.R., Zunc I. (2006). Characterization of diabatic two-phase flows in microchannels: Flow parameter results for R-134a in a 0.5 mm channel. Int. J. Multiph. Flow.

[19] Unger M.A., Chou H., Thorsen T., Scherer A., Quake S.R. (2000). Monolithic microfabricated valves and pumps by multilayer soft lithography. Science.

[20] Utada A.S., Lorenceau E., Link D.R., Kaplan P.D., Stone H.A., Weitz D.A. (2005). Monodisperse double emulsions generated from a microcapillary device. Science.

[21] Hassan S., Morgan H., Zhang X., Niu X. (2015). Droplet interfaced parallel and quantitative microfluidic-based separations. Anal. Chem..

[22] Karmali K., Karmali A., Teixeira A., Curto M.J.M. (2004). Assay for glucose oxidase from *Aspergillus niger* and *Penicillium*
*amagasakiense* by Fourier transform infrared spectroscopy. Anal. Biochem..

